# The Reliability of Auto-Injectors in Clinical Use: A Systematic Review

**DOI:** 10.7759/cureus.41601

**Published:** 2023-07-09

**Authors:** Petr Dostal, Jorg Taubel, Ulrike Lorch, Vishal Aggarwal, Thomas York

**Affiliations:** 1 Clinical Research, University of Cambridge, Cambridge, GBR; 2 Cardiology, Richmond Pharmacology Ltd., London, GBR; 3 Anaesthesiology, Richmond Pharmacology Ltd., London, GBR; 4 Clinical Research, Richmond Pharmacology Ltd., London, GBR

**Keywords:** delivery, drug, pharmacology, pharmaceutical, clinical development, clinical trial, injection, auto-injector

## Abstract

Auto-injectors are medical devices designed for the self-administration of injections by patients and for easy administration by healthcare professionals in emergency situations. Although they vary in design and application, auto-injectors are typically built around a spring-loaded syringe. Despite their widespread use in a variety of clinical settings, there have been limited attempts to assess their reliability. This systematic review investigates the reliability of auto-injectors, identifies common causes of failure, and summarizes the overall rate of malfunction. A systematic review of research published on the PubMed and Cochrane Library databases was performed in July 2022. The relevant studies were assessed for their methodological quality and risk of bias prior to extracting key study outcomes on auto-injector reliability. Finally, a summary rate covering all eligible studies was calculated. The search identified a total of 110 articles, of which ten were found to be suitable for inclusion. The risk of bias was low, and the methodological quality was high across the ten studies. Out of a total of 2,964 injections administered from an auto-injector, there were 12 device malfunctions, giving a summary rate of 0.40% (±0.23) auto-injector failures. The causes of malfunction varied in nature, with the majority of cases (58.3%) not being specified or not identified. This review has demonstrated that auto-injectors are reliable devices. Although further research on the nature of malfunctions is needed, the low rate of malfunctions supports training programs for healthcare professionals and patients on the optimum use and maintenance of auto-injectors. It provides a rationale for their continued development.

## Introduction and background

Auto-injectors are spring-loaded syringes designed for self-administration by patients and easy administration in emergency situations, such as anaphylaxis, by healthcare professionals. Given that the device contains pre-measured medication, this eliminates the need to draw up medication from a separate vial. Moreover, this allows people without formal medical training to use auto-injectors [1]. There are two primary types of auto-injectors: prefilled syringe-based systems and cartridge-based systems [2]. Both can administer medication via the subcutaneous (SC) or intramuscular (IM) route. Auto-injector technology was originally developed for military personnel to enable rapid and reliable delivery of life-saving antidotes, such as atropine, in high-stress battlefield situations as an antidote to weaponized gases [1]. Later, auto-injector technology was utilized in the development of the EpiPen, an auto-injector prefilled with a dose of adrenaline for the quick treatment of anaphylaxis. The EpiPen was approved by the FDA in 1987. Since then, there has been a large growth in the use of auto-injectors for drug administration, including their use outside of emergency situations. As of August 2021, almost 80 auto-injectors have been developed by over 20 pharmaceutical companies [3]. These include atropine for nerve agent poisoning, epinephrine for anaphylaxis, monoclonal antibodies for chronic diseases such as rheumatoid arthritis or systemic lupus erythematosus, diazepam for seizures, sumatriptan for migraines, and more [4]. This demonstrates a wide range of auto-injectors used across a variety of clinical purposes. The introduction of auto-injectors for treating chronic diseases, in addition to emergencies, has improved the quality of patient care. Auto-injectors are simple to use, overcome hesitance, cause less pain at the injection site, and lead to improvements in adherence. As a result, patients using auto-injectors are more satisfied and prefer them to alternatives [5,6]. Auto-injectors have been beneficial to patients requiring SC/IM medication administration, justifying the uptake in utilization. Despite their widespread adoption, there have so far been limited attempts to assess the reliability of auto-injectors. More specifically, there is insufficient data on the mechanisms for malfunction. The FDA defines the reliability of a manufactured product as the probability that it will perform satisfactorily for a specified period of time under stated use conditions. Not performing satisfactorily implies the failure of one or more product components. In all manufactured products, there is a measure of reliability called 'failure rate' [7]. The recommendation exists that emergency-use injectors should meet design control 177 specifications, ensuring a successful injection reliability rate of 99.999% with a 95% level of confidence. This study is a systematic review of the published, peer-reviewed literature on the reliability of such devices. It presents novel information on common causes for device failure and assesses the implications of this on auto-injector development, laying the foundation for further exploration into this delivery modality.

Methods

Review Protocol and Search Strategy

A thorough scoping search revealed no similar works to this paper at the time of writing. PubMed MEDLINE and Cochrane Library databases: a. Cochrane Database of Systematic Reviews, b. Cochrane Central Register of Controlled Trials was scrutinized from 1990 to July 2022 using search criteria covering two sets of key terms: (1) Auto-injector: auto-injector, auto-injector, auto-injection, self-injector, spring-loaded syringe; (2) Reliability: error, fail, fault, break, early activation, leak, malfunction. The search combined terms for auto-injectors with terms that could be used to describe reliability issues, as informed by the native-English standard.

Inclusion Criteria

With the objectives of the literature review in mind, the authors developed eligibility criteria to identify articles suitable for inclusion in the review. The criteria were: (1) articles needed to be published in English, even those that have undergone translation; (2) pertain to human rather than veterinary medicine; (3) Studies conducted during or after 1990 were included, with this year chosen as the starting point because the EpiPen was registered in 1987 and 1990 was the earliest searchable year. Additionally, the full text of the article needed to be available for review. The subject of study should feature auto-injectors, defined for this review as a device capable of delivering a metered dose of an injectate without manual depression of a syringe plunger. In other words, the manual application of standard activation energy to a switch should cause the device to deliver an actuation force to the injectate, thereby delivering the injection. As a result, devices such as insulin pens were excluded since their mechanism of delivery involves manually depressing a plunger, the depth of which is dependent on the dose selected. Furthermore, the data needed to be generated by injection into human tissue or a suitable analog, such as a foam pad mimicking human skin behavior. Lastly, auto-injectors that were described as novel or already registered, such as the EpiPen, were included.

Exclusion Criteria

The exclusion criteria for the review included data generated by techniques that do not model injection into human tissue, such as activation of an auto-injector into empty space; auto-injectors described as prototypes or preliminary versions; and devices like certain insulin pens because their mechanism of delivery involves manually depressing a plunger, the depth of which is dependent on the dose selected. For the purposes of this review, auto-injectors were defined as a device capable of delivering a metered dose of an injectate without manual depression of a syringe plunger.

Study Selection and Data Collection

After identifying records by the described search strategy, each abstract was independently examined against the eligibility criteria by the review authors (Petr Dostal and Thomas York). In instances where the authors reached differing conclusions, the report was collaboratively discussed until a consensus on its inclusion was reached. The data required to assess the rate of malfunction was extracted from each included paper by the independent, manual review of Petr Dostal and Thomas York. The rate obtained was verified between the two reviewers prior to finalization for inclusion in this report. Risk of bias and methodological quality assessment was conducted by Petr Dostal and subsequently reviewed by Thomas York.

Data Assessment

An assessment was made of methodological quality using a modified MINORS tool [8] and of risk of bias using a modified Cochrane RoB2 tool [9]. The MINORS tool includes nine methodological items for non-randomized studies and four additional items for a comparative study. Since not all of the studies selected were comparative, the nine-item version of the tool was applied. Similarly, the Cochrane RoB2 tool includes five items, but the first item, "Risk of bias arising from the randomization process," was not applicable due to the included literature being made up of single-arm studies. Consequently, a four-item, modified version was used to assess the risk of bias. After the assessment, individual malfunction rates were extracted from the papers, and a comprehensive summary rate was calculated.

## Review

Results

Study Selection and Data Collection

**Figure 1:**
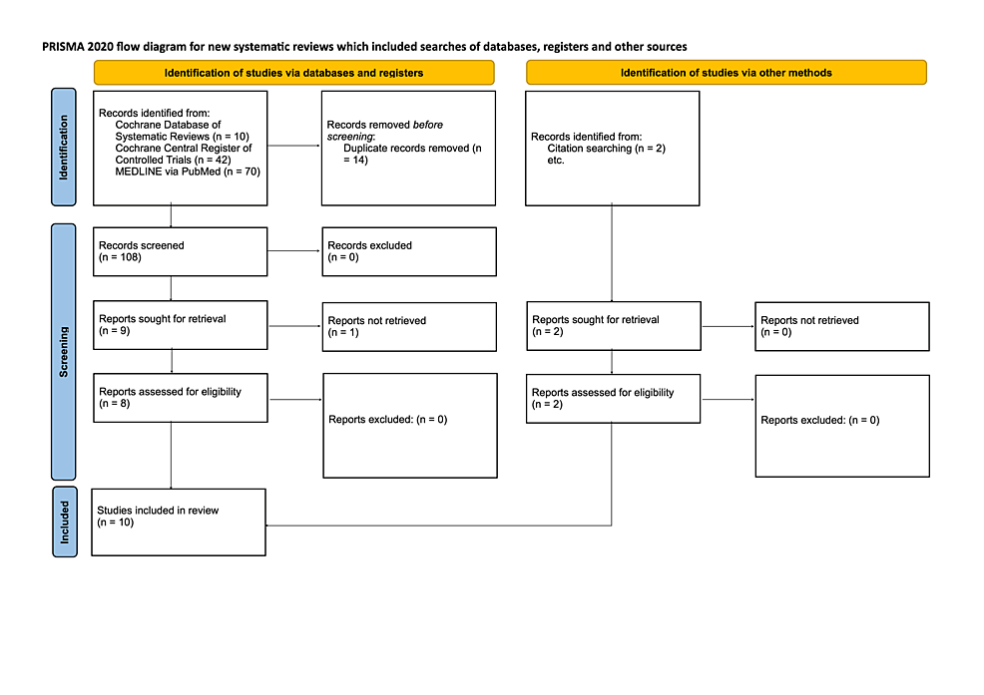
The literature review process.

The search identified a total of 108 articles following the removal of duplicates (Figure 1). In accordance with Preferred Reporting Items for Systematic Reviews and Meta-Analyses (PRISMA) guidelines, abstracts of these articles were reviewed, with eight progressing to full-text review. Following a full-text review, all eight of these articles were found to be suitable for inclusion. During the abstract review, a second article by Kaestner et al. contained data that may have been relevant. After extensive attempts, the authors were unable to obtain full-text access to the article. Consistent with eligibility criteria, they were excluded from the subsequent systematic review [10-18].

A process of citation-chaining, reviewing references for further potentially eligible works, was performed on the eight included articles. This identified an additional two articles [19-20]; after abstract and full-text review, both were found to be eligible for inclusion. This resulted in a total of 10 articles being included in the review.

Risk of Bias Assessment

A modified Cochrane risk of bias tool [9] was used to assess the selected studies for risk of bias on four separate criteria (Table 1). Overall, most studies (6/10) were considered low risk, and none were considered high risk [12,14-17,20]. The studies considered to have "some concern" [11,13,18-19] over the risk of bias (4/10) were assessed as such due to outcomes being measured and reported by the participants rather than external observers. Our assessment concluded an overall low risk of bias across the studies included in this review.

**Table 1:** Modified Cochrane risk of bias assessment tool.

Study	Risk of bias due to deviations from the intended interventions	Missing outcome data	Risk of bias in measurement of the outcome	Risk of bias in selection of the reported result	Overall risk of bias
Sheikh SZ et al. (2016) [10]	Low risk	Low risk	Some concerns	Low risk	Some concerns
Struemper H et al. (2016) [13]	Low risk	Low risk	Low risk	Low risk	Low risk
Xu Z et al. (2015) [11]	Low risk	Low risk	Low risk	Low risk	Low risk
Phillips JT et al (2011) [12]	Low risk	Low risk	Some concerns	Low risk	Some concerns
Schwarzenbach F et al. (2014) [14]	Low risk	Low risk	Low risk	Low risk	Low risk
Guerlain S et al. (2010) [15]	Low risk	Low risk	Low risk	Low risk	Low risk
Martin UJ et al. (2021) [16]	Low risk	Low risk	Low risk	Low risk	Low risk
Ferguson GT et al. (2019) [18]	Low risk	Low risk	Some concerns	Low risk	Some concerns
Alpizar S et al. (2021) [17]	Low risk	Low risk	Some concerns	Low risk	Some concerns
Zheng Y et al. (2021) [19]	Low risk	Low risk	Low risk	Low risk	Low risk

Methodological Quality

The selected studies were assessed for their methodological quality using a modified MINORS tool [8]. Out of a possible score of 16, the range of scores was 10-15 (Table 2). The scores were high across the studies, with an average score of 13.8. This supports a high degree of methodological quality in the studies included in this review.

**Table 2:** The methodological quality assessment using a modified MINORS assessment tool.

Study	Clearly stated aim	Inclusion of consecutive patients	Prospective collection of data	Endpoints appropriate to the aim of the study	Unbiased assessment of the study endpoint	Follow-up period appropriate to the aim of the study	Loss to follow up less than 5%	Prospective calculation of the study size	Total
Sheikh SZ et al. (2016) [10]	2	2	2	2	2	2	2	0	14
Struemper H et al. (2016) [13]	2	2	2	2	2	2	2	1	15
Xu Z et al. (2015) [11]	2	2	2	2	2	2	2	1	15
Phillips JT et al (2011) [12]	1	2	1	1	2	0	1	2	10
Schwarzenbach F et al. (2014) [14]	2	1	2	2	2	2	2	2	15
Guerlain S et al. (2010) [15]	2	1	2	2	2	2	2	0	13
Martin UJ et al. (2021) [16]	2	2	1	2	2	2	2	1	14
Ferguson GT et al. (2019) [18]	2	1	2	2	2	2	2	1	14
Alpizar S et al. (2021) [17]	1	2	2	2	2	2	2	0	13
Zheng Y et al. (2021) [19]	2	2	2	2	2	2	2	1	15

Outcomes

Across the 10 studies, nine of which utilized subcutaneous administration, 809 participants were included with 2,964 injections given from an auto-injector (Table 3).

**Table 3:** Summary of findings. SC: Subcutaneous; IM: Intramuscular.

Study	Number of Participants	Method	Number of injections given via an auto-injector	Number of auto-injector malfunctions	Malfunction rate /%
Sheikh SZ et al. (2016) [10]	91 out of 95 systemic lupus erythematosus patients completed the study	Participants self-administered belimumab SC using an auto-injector weekly for 8 weeks.	736	2 (a)	0.27
Struemper H et al. (2016) [13]	41 healthy volunteers	Participants self-administered a single SC belimumab injection by an auto-injector	41	1 (b)	2.44
Xu Z et al. (2015) [11]	77 healthy male subjects	Participants received a single SC golimumab injection using the SmartJect auto-injector	77	0	0.00
Phillips JT et al (2011) [12]	71 out of 74 multiple sclerosis patients completed the study	Participants self-administered weekly IM IFNβ-1a injections using the Avonex Pen™ for 4 weeks	215	0	0.00
Schwarzenbach F et al. (2014) [14]	65 rheumatoid arthritis patients	Participants performed 6 SC injections using the BD Physioject auto-injector and a foam pad that mimicked skin behaviour	390	0	0.00
Guerlain S et al. (2010) [15]	48 participants	Participants performed simulated-use testing using trainer models of EpiPen and TwinJect in an anaphylaxis scenario	96 (48 with each device)	1 (c)	1.04
Martin UJ et al. (2021) [16]	90 healthy adult men	Participants received a single SC benralizumab injection via using an auto-injector	90	0	0.00
Ferguson GT et al. (2019) [18]	121 patients with severe asthma	Participants received benralizumab SC injections using an auto-injector at weeks 0, 4, and 8 at the study site. They received the first dose from a healthcare professional, had the option to self-administer the second dose, and had to self-administer the third dose under supervision. Participants self-administered at home at weeks 12 and 16.	595	2 (d)	0.34
Alpizar S et al. (2021) [17]	105 patients with uncontrolled asthma	Participants received 6 SC tezepelumab injections using an auto-injector, with the first dose being given by a healthcare professional and the subsequent doses being administered by the participants or their carers.	624	5 (e)	0.80
Zheng Y et al. (2021) [19]	107 healthy volunteers	Participants received a single SC tezepelumab dose via an auto-injector from trained study staff.	107	1 (f)	0.93
Total / Absolute malfunction rate	816 participants completed the studies		2971	12	0.40

Four of these studies [12-13,15,17] reported no malfunctions with the auto-injector devices, while the other six reported at least a single malfunction (Table 3). The causes of malfunction were divided into four categories (Table 4). In most cases (58.3%), the nature of the device failure was either not specified or not identified. Failure to deliver due to user error was not included; neither data nor observations were outlined in the studies regarding this, and the patients received standardized training beforehand. ​​​​

**Table 4:** Summary of reasons for auto-injector malfunctions.

Reason for auto-injector failure	Frequency	Percentage of total (%)
Auto-injector did not activate	2	16.7
Failed to deliver the injection fluid	2	16.7
Safety cap came off early	1	8.3
Not specified or not identified	7	58.3

Out of a total of 2,964 injections, there were 12 device malfunctions, giving a summary rate of one malfunction for every 247 injections (mean=0.40 ±0.23%). These malfunctions took various forms. In one case, the delivery stopped before the end of the injection, and in another, the auto-injector did not deliver any injectate at all. There were instances where the auto-injector failed to activate, potentially due to the drying of the needle tip with the drug product causing a blockage or a higher-than-usual activation force due to a minor component defect observed by X-ray. However, specificities pertaining to these defects were not mentioned. In another instance, the safety cap came off simultaneously with the outer case on an EpiPen. One of the auto-injectors malfunctioned because of a manufacturing defect, while the cause of the other malfunction could not be definitively identified. For some devices, the reasons for malfunctions were not specified, but no mechanical or design-related issues were found. There was also a case where all injection fluid remained within the device.

Discussion

Auto-injectors are devices designed to allow easy administration of medication via the SC and IM routes. With the growing use of auto-injectors for the treatment of various medical conditions, this literature review aimed to assess the reliability of auto-injectors and the mechanisms for device malfunction. Scoping searches revealed that there have so far been very limited efforts to synthesize summary information on such a topic. The generation of such data is critical to providing information on the reliability of auto-injector technology. It has been more widely identified as a key component in medical device development [21]. For the propagation of auto-injectors across clinical practice, it is essential to see that they can be used with a minimal incidence of malfunction, and while the expansion of patient education programs will be a key factor in the technology's uptake, a review of such programs is beyond the scope of this paper.

Eligibility Criteria

Auto-injectors were defined as a device capable of delivering a metered dose of an injectate without manual depression of a syringe plunger. While nuances exist in the definition of an auto-injector, the above interpretation represents the most widespread understanding. Frew AJ [2] described the auto-injector device as needing a "thumb activation," following which the "released spring moves the prefilled syringe to its end position," outlining the use of standard activation energy. Additionally, Xu Z et al. [11] described the auto-injector as "a prefilled, spring-powered" similar to Phillips JT et al. [13], who used an auto-injector with a "two springs" which were activated by a "depressing blue activation button." Therefore, our definition of an auto-injector was consistent with a widely accepted one. Auto-injector devices described as "prototype" were excluded as these auto-injectors did not verifiably meet the manufacturing standard required for clinical use [22]. In these cases, it was also impossible to confirm that the mechanism of the prototype auto-injector was consistent with the definition used in this review. These eligibility criteria have the potential of excluding relevant research. However, it was considered that their application was necessary to standardize the search and achieve an understanding of auto-injectors relevant to actual clinical practice.

Data Assessment

The selected studies were assessed for risk of bias using a modified Cochrane risk of bias (RoB2) tool [9] and for their methodological quality using a modified MINORS tool [8]. As outlined in the Results section, the studies that were reviewed were reliable and suitable for inferring a conclusion as they were assessed to have an overall low risk of bias and a high degree of methodological quality. The findings of the assessment were reassuring as they suggested that the studies and their findings were reliable and suitable for inferring a conclusion.

Review Findings

As of the writing of this review, the reliability of auto-injectors has not been well assessed. Following an assessment of the 10 studies that were suitable for this review, auto-injectors have been found to be generally reliable as only 12 malfunctions from 2,071 injections were reported, giving a summary rate of 0.40%. To set this against the standard of other medical devices, Marsh N et al. [22] have shown that peripheral IV catheters, widely used medical devices that play an essential role in the care of patients, have a failure rate of 36%. Such a low number of auto-injector malfunctions suggests that the devices are generally reliable, and given that patients have shown a preference for their use over conventional syringes [5-6], there is a clear incentive to continue the development of this technology and apply it to an even broader range of purposes. It should, however, be considered that such low rates of reported malfunction may be indicative of systemic under-reporting. Vicente KJ et al. record this phenomenon, citing the influence of insufficient and poorly understood reporting mechanisms; 94.2% of respondents were unaware of the national, medical device problem report form [23]. This literature review also aimed to explore the reasons for device malfunctions. Three different reasons were established to be the cause of five of the 12 failures: failure to activate, failure to deliver complete injectate volume, and the safety cap separating from the device prematurely. One of the failures was concluded to be due to a manufacturing defect though the defect was not specified. These findings suggest targets for more in-depth study to assess device malfunction mechanisms, improve manufacturing techniques, and optimize usability. This is already a clear commercial focus for manufacturers [24-26] and should also be made central to the clinical selection of appropriate auto-injector devices. Finally, the reasons for the other seven failures were not described or not established. The lack of quality in this reporting presents an issue in assessing overall auto-injector safety and reliability. Furthermore, there could be a systemic issue with performing in-depth evaluations of device malfunctions, as the approval process for medical devices is less stringent than for drugs [27]. Nevertheless, it is essential to differentiate reporting in the peer-reviewed literature from the compulsory reporting submitted to regulatory bodies such as the FDA's MedWatch [28] or the Medicines and Healthcare Products Regulatory Agency (MHRA)'s Yellow Card Scheme [29]. When comparing auto-injectors against conventional syringes, it could be argued that additional device complexity inherently predisposes to malfunction, making an auto-injector more prone to failure than a simple syringe. This is a logically tempting principle to apply. However, the complexity of medical devices is a complex topic [30], and improvements in design have shown that intricate manufacturing requirements do not necessarily lead to complexity for the end user [30]. Despite the increased complexity of auto-injectors, studies such as those from Ziemssen T et al. [5], Berteau C et al. [6], and Pozzilli C et al. [7] have made clear that patients prefer them. This leads to benefits such as improved compliance which in turn optimizes patient care. Moreover, this literature review has found that the rate of auto-injector malfunctions is low (0.40%), despite their complexity. While a direct comparison between conventional syringes and auto-injectors is challenging, the factors described here suggest a favorable environment for developing the technology.

Limitations

One limitation of this review is that a relatively low number of studies were found to be eligible for inclusion. Using a small sample increases the chance that a false premise is assumed to be true [31]. Having said that, a small number of studies on auto-injectors have reported device malfunctions, supporting their reliability.

Future Development

Human factors are not sufficiently considered during the safe development of a medical device [32], such as an auto-injector. Despite their reliability, the auto-injectors will not deliver the injectate if the user makes an error due to inadequate usability. A systematic review by Weinhold T et al. [32] has found a significant number of user errors related to auto-injectors that could be classified into a broad range of categories. This can serve as guidance for the future development of auto-injectors to ensure good usability alongside their reliability.

## Conclusions

This review has demonstrated the reliability of the auto-injectors, providing a rationale for the increased growth in their use. While the rate of failures is low, further research is warranted to assess the mechanisms behind them.
